# Application of Nitrate of Silver to the Larynx in Croup

**Published:** 1848-04

**Authors:** Charles E. Ware

**Affiliations:** Boston


*Application of the Nitrate of Silver to the Larynx in Croup.* [Read before the Boston Medical Society for Improvement, by Charles E. Ware, M.D.]—The very great mortality which attends membranous croup, makes every case of recovery interesting, and the treatment, when it has been consistent and efficient, instructive. Although we frequently hear well-informed practitioners talk flippantly of their success in the management of croup, yet every one who is in the habit of discriminating between the two forms of the disease, knows with what a sinking heart and faltering hand he undertakes the treatment of the one, and with what comparative confidence he deals with theother.

Within the last two or three years, the system of steaming by the inhalation of hot vapors of herbs of various kinds, has been tried pretty extensively in this neighborhood, and I should think from the number of successful cases reported before this Society, with encouraging success. One great and most serious objection, however, to this treatment, supposing its value established, is in many cases the impossibility, and in all the extreme difficulty, of applying the remedy effectually, with any apparatus which has yet been devised, and ingenuity in and out of the profession has not been idle in contriving. This obstacle in the way of the treatment which would seem to present the most promise, together with some apparent success in the applications of the nitrate of silver to acute inflammation of the tonsils both in children and adults, and the facility with which the nitrate of silver may be applied to the larynx with but trifling irritation in the adult, led me lately to try the application of it to the larynx of a child 5 years old, laboring under unequivocal membranous croup.

I was called to this patient, a boy 5 years old, Saturday, November 20th. The mother said that he began to breathe hard just a week previous, but as he had been subject to attacks of spasmodic croup, in several of which I had attended him, and usually found ready relief, she felt no great anxiety, especially as there was less constitutional affection than he had had in former attacks. His tonsils, also, had been for a year or more somewhat enlarged, and often gave a huskiness to his respiration and voice. On Tuesday he began to cough, and evinced other signs of a cold. These symptoms continued, he playing about, and having appetite, without anything very characteristic, till Friday afternnoon, when the cough began to have a ringing tone, and the respiration to be very labored. I was not called to him till the next afternoon. Then there was the characteristic breathing of croup well marked. It had become sufficiently distressing to occasion great restlessness and jactitation, but was not accompanied by much febrile excitement, nor, as yet, prostration. The general expression of the child was good. On examining the fauces, they appeared red, and the tonsils presented distinct patches of lymph. On the backs there was an almost entire absence of respiratory sound, and no râles whatsoever. The breathing and cough were both dry, with very little râle in the trachea.

He was ordered strong mercurial ointment and a fomentation to neck, and pills of Dover's powder and blue pill once in four hours, and a syrup with opium and ipecac. at intervals between. Under the influence of the opium he got more sleep than during the previous twenty-four hours, but in the morning was no better, nor essentially different.

I now commenced the application of the nitrate of silver to the larynx, using a solution of the strength of a drachm to the ounce, and applying it with one of Dr. Green's whalebone staffs. I applied it twice in the course of the first day. The child the first time resisted violently, and I was obliged to use much force. But after having been persuaded once to submit quietly, the operation occasioned so little irritation that he never afterwards made the least objection to it, but allowed me to perform it as effectually as I could have done upon a grown person. The first applications were followed by so much excitement that it was difficult to see what was the immediate effect. But afterwards, when he was more tranquil during the operation, it was obvious that it produced an increased dryness of the cough and respiration, without immediate relief or aggravation of the labor in breathing. In the course of the day he raised twice considerable pieces of false membrane, very well marked, and stained with blood, together with a great deal of very thick, tenacious mucus, which was also occasionally stained with blood. After the discharge of the false membrane, the breathing became much easier, and was never again as labored, as it had been before.

The next day, Monday, respiration, although improved, was still very laborious. There was yet great deficiency of respiratory sound in the back, and absence of râles. After the application of the caustic, which was applied twice this day, the fauces appeared red, but there was no lymph visible. From this time the amendment, although slight, from day to day was constant, till Friday, the 26th. No lymph was seen upon the tonsils. The caustic was applied once a day. Friday night, the breathing was more labored than the night before, and Saturday morning I again saw lymph upon the tonsils. Through Saturday and Sunday, however, he continued to improve, and Sunday evening uttered the first loud word which he had been able to speak since I had seen him. The caustic was now omitted, as well as all his medicines. His appetite, which had never entirely disappeared, became more urgent, and he was allowed to eat freely. Indeed his diet had been liberal throughout. The respiratory sound gradually returned to the backs, but continued as it had done throughout, free from râle. The voice continues to improve, but still retains a huskiness. The caustic was applied twice the two first days; aftewards but once a-day, the sponge never being introduced more than once at the same visit.

This is a case of croup in which the disease was obviously limited to the trachea, if not to the larynx, and in this respect was as favorable for the application of the peculiar treatment as it could be. In the majority of cases, from my own observation, the disease extends more or less into the lungs.

Of five *post-mortems* which I have made, in one case disease was limited to to the larynx and upper part of the trachea. In the other four it extended far into the lungs, in one producing chronic pneumonia, of which the child died after recovering from the croup. In such cases one would not expect much from the treatment, since the application could only be made over a very small extent of the diseased surface. It is as yet, however, not very well understood precisely where the obstruction takes place, which occasions the death in croup. It is probable that it varies greatly in different cases. I have seen once or twice, in *post-mortem* examinations, the trachea and larynx so thinly coated, and to such a limited extent, that it seemed impossible that it should not have been sufficiently free for respiration, while the large bronchi of the lungs on both sides were almost entirely obstructed by thick tenacious mucus. In such cases the source of death is probably obstruction of the bronchi. But from the great relief which is almost always given by the rejection of pieces of false membrane, and from the frequency of its occurrence (indeed, since attention has been drawn to the point, I think there are few cases occur in which it is not observed), it is probable that the cause of death is generally in the trachea, and most frequently at the upper part, or in the larynx. The latter fact would be indicated by the almost universal, if not universal, temporary relief which the operation of tracheotomy gives in these cases. If this part, then, could be kept free, the chances would be greatly increased of the child's being able to struggle against the disease.

The cases in which I have applied the nitrate, or seen it applied, to inflammatory affections of patients in which there was a tendency to this deposit upon the mucus membrane, have been as yet few, but it has certainly appeared that the application had much efficiency in keeping the membrane clear.

This is one case of recovery. My own observation would furnish nothing more in favor of any other treatment. Of fourteen cases of croup which I have either attended myself or seen during their course, four have recovered. In one of the cases of recovery, there was no treatment. The child was so violent that nothing could be done. The disease was left literally to its natural course. In the second case the treatment was essentially opiate. In the third case, the steaming was applied more efficiently than I have ever seen it in any other case. The fourth case was the present. An interesting point in this case was the speedy recovery of the voice. The child spoke loud at the end of a week after the treatment commenced. In all other cases of recovery which I have known, the absence of the voice has continued for many weeks; five or six has, I think, been the shortest limit.

The case is valuable, also, as showing that a sponge saturated with so strong a solution of nitrate of silver, can be introduced into the larynx of a child not only with safety, but, after the first application, with so little inconvenience.—*Boston Med. and Surg. Journal.*

Since reading the above case, an article by Dr. Blakeman, of New York, in the American Quarterly retrospect, has been pointed out to me, according to which he has employed this same treatment with success.

Boston, Dec. 16, 1847.

